# Beyond Hot Flashes: The Role of Estrogen Receptors in Menopausal Mental Health and Cognitive Decline

**DOI:** 10.3390/brainsci15091003

**Published:** 2025-09-16

**Authors:** Jung Min Cho, Jihye Lee, Eun-Mi Ahn, Jaehoon Bae

**Affiliations:** 1 Functional Food Research Institute, Industry-University Cooperation Foundation, Daegu Haany University, Gyeongsan-si 38610, Republic of Korea; jungmincho@dhu.ac.kr (J.M.C.); ahnem@dhu.ac.kr (E.-M.A.); 2 Hybrid Cosmetics Research Institute, Industry-University Cooperation Foundation, Daegu Haany University, Gyeongsan-si 38610, Republic of Korea; jhlee86@dhu.ac.kr; 3 Department of Food Science and Biotechnology, Daegu Haany University, Gyeongsan-si 38610, Republic of Korea

**Keywords:** cognitive decline, estrogen receptors, menopause, mental health, neuroprotection

## Abstract

Menopause is a natural phase in a woman’s life marked by the cessation of menstruation, typically accompanied by hormonal fluctuations that have significant impacts on physical and mental health. While much attention has been given to the physical symptoms of menopause, such as hot flashes and osteoporosis, the neurocognitive consequences of hormonal fluctuations during the menopausal transition and the subsequent sustained estrogen loss after menopause have received less focus. Estrogen receptors (ERs), specifically ERα and ERβ, play a critical role in maintaining brain health, influencing mood, memory, and cognition. This review explores the connection between estrogen receptor signaling and mental health during menopause, focusing on mood disorders such as depression and anxiety, as well as cognitive decline and dementia. We discuss the molecular mechanisms by which ERs modulate brain function, including their effects on neuroplasticity, neurotransmitter systems, and gene expression. The review also examines current clinical approaches to managing menopausal cognitive and mental health issues, including hormone replacement therapy and selective ER modulators, while emphasizing the need for further research into alternative therapies and individualized treatments. The importance of estrogen receptors in the menopausal brain and their potential as therapeutic targets is critically evaluated, aiming to shed light on this often-overlooked aspect of menopause and aging.

## 1. Introduction

The menopausal transition is characterized by fluctuating estrogen levels that precede the final menstrual period, whereas menopause is retrospectively defined after 12 consecutive months without menstruation and is associated with a sharp and sustained decline in estrogen production. Characterized by a decrease in ovarian estrogen production, menopause typically occurs between the ages of 45 and 55, resulting in profound changes in a woman’s physical, emotional, and cognitive well-being [1]. While hot flashes, vaginal dryness, and osteoporosis have long been the hallmark concerns of menopausal women, the neurocognitive consequences of estrogen depletion remain underexplored. As women age, the risks of mental health disturbances, including depression, anxiety, and cognitive decline, increase substantially. Notably, alterations in the central nervous system may emerge before the onset of vasomotor symptoms, suggesting that cognitive and emotional changes can precede hot flashes and other physical manifestations. These conditions not only diminish the quality of life but also contribute to the growing burden of age-related diseases in the aging population [2].

Estrogen, primarily through its interaction with estrogen receptors (ERs), plays a critical role in the brain, exerting effects on a variety of physiological processes, including mood regulation, cognitive function, and synaptic plasticity [3]. ERs, namely estrogen receptor alpha (ERα) and estrogen receptor beta (ERβ), are widely expressed in areas of the brain associated with emotion and cognition, such as the hippocampus, prefrontal cortex, and amygdala [4,5,6]. Their presence in these brain regions suggests that estrogen has a significant impact on neural activities, particularly in the regulation of mood and memory [7]. With the dramatic decline in estrogen levels that occurs during menopause, these receptors become less active, which may explain the onset of cognitive decline and mood disorders that many women experience during this period [8].

Despite the growing recognition of these issues, the relationship between ER activity and menopausal mental health has received limited attention compared to the well-documented effects of estrogen on physical symptoms. The neuroprotective roles of estrogen, its influence on neurotransmitter systems such as serotonin and dopamine, and its modulation of synaptic connections in the brain all contribute to its ability to support cognitive function and emotional well-being [9]. However, the complex interplay between ERs and other molecular pathways, including inflammatory processes, oxidative stress, and neurogenesis, remains poorly understood.

This review aims to bridge this gap by critically examining the role of ERs in menopausal mental health and cognitive decline. We explore the molecular mechanisms through which ERs mediate the effects of estrogen on the brain and discuss the clinical implications of these findings. In particular, we highlight the potential of estrogen receptor modulators, hormone replacement therapy (HRT), and alternative treatments in improving mental health and cognitive outcomes in menopausal women. By shedding light on these underexplored areas, this paper seeks to promote a more comprehensive understanding of menopause and emphasize the importance of considering mental health and cognitive function alongside physical symptoms in managing menopausal health. The literature search and selection process was conducted according to PRISMA guidelines, and the overall flow of records through identification, screening, and inclusion is presented in Supplementary Figure S1.

## 2. Estrogen Receptors in the Brain

**Figure 1:**
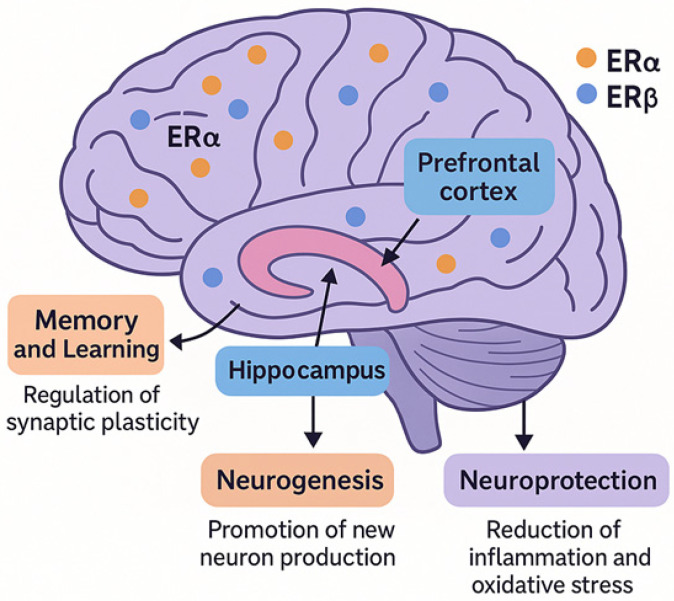
Estrogen receptors in the brain and their functional roles. Distribution and function of estrogen receptor alpha (ERα) and estrogen receptor beta (ERβ), the two main subtypes of estrogen receptors in the brain. Both receptors are widely expressed, with ERα particularly enriched in the hippocampus and prefrontal cortex, regions critical for learning, memory, mood regulation, and executive function. Estrogen, through these receptors, promotes synaptic plasticity, neurogenesis, and neuroprotection. It also modulates neurotransmitter systems (e.g., serotonin, dopamine, glutamate), reduces inflammation and oxidative stress, and supports cognitive and emotional health, especially before menopause. The decline of estrogen during menopause contributes to cognitive impairments and increased risk of neurodegenerative disorders.

Estrogen, primarily through its interaction with ERs, exerts significant influence on brain function, particularly in regions involved in cognition, mood, and memory. The two main types of ERs, ERα and ERβ, are both widely expressed throughout the brain, with their distribution varying by brain region [10]. Notably, ERα is found in high concentrations in areas associated with memory and learning, such as the hippocampus and prefrontal cortex [11,12]. These brain regions are pivotal in regulating both emotional responses and cognitive functions, suggesting that estrogen, through ERα and ERβ, plays a central role in supporting neural processes essential for memory formation, mood regulation, and decision-making [13] (Figure 1).

The hippocampus, an area critically involved in learning and memory processes, is particularly sensitive to estrogen. Research has shown that the presence of ERα in this region is closely linked to the maintenance of synaptic plasticity, the ability of synapses to strengthen or weaken over time, which is a fundamental mechanism underlying learning and memory [14]. Estrogen, via ERα, has been demonstrated to promote neurogenesis in the hippocampus, stimulating the production of new neurons and enhancing their connectivity [15,16,17]. This neuroprotective role is crucial during aging, as the decline of estrogen levels in postmenopausal women can lead to the loss of this protective effect, thereby contributing to cognitive decline [18]. The loss of estrogen’s neuroprotective and neurogenic effects during menopause is implicated in an increased risk of age-related neurodegenerative diseases, including Alzheimer’s disease [19].

In addition to the hippocampus, ERs are expressed in the prefrontal cortex, a brain region responsible for higher cognitive functions, including working memory, executive control, and decision-making. Estrogen’s influence on ERα in the prefrontal cortex modulates the strength of synaptic connections and neuronal excitability, enhancing cognitive flexibility and processing speed [13,20,21]. This modulation is particularly important in the context of aging, as declines in estrogen during menopause are associated with impairments in executive functions, which can manifest as difficulties in memory recall, multitasking, and problem-solving [22,23].

Beyond structural changes, estrogen also affects neurotransmitter systems in the brain, which play a central role in regulating mood and cognition [14]. One of the most studied neurotransmitter systems influenced by estrogen is the serotonin system. Studies have shown that estrogen upregulates serotonin receptor expression, particularly in the hippocampus and the prefrontal cortex, areas crucial for emotional regulation. Through this mechanism, estrogen helps to maintain a balanced mood and is thought to have an antidepressant-like effect [22,24,25]. The decline in estrogen during menopause, therefore, can exacerbate mood disorders such as depression and anxiety, as reduced serotonin activity becomes a key contributor to these conditions [26,27].

Estrogen also interacts with the dopaminergic system, which is integral to motivation, reward processing, and cognitive flexibility [28]. ERα activation in the basal ganglia and other dopaminergic regions of the brain has been associated with improvements in reward-seeking behaviors and cognitive performance [29]. In menopausal women, the reduction in estrogen can impair dopamine signaling, contributing to decreased motivation, anhedonia, and difficulties with cognitive tasks that require adaptive responses. Additionally, the role of estrogen in the modulation of glutamate, the primary excitatory neurotransmitter, has implications for synaptic plasticity and learning [26,30]. Glutamate signaling, which is regulated by ERs, is crucial for maintaining the brain’s ability to respond to new experiences and store memories [17].

While much of estrogen’s action in the brain is mediated by genomic mechanisms, which involve direct regulation of gene expression through estrogen receptor binding, non-genomic signaling pathways have also been implicated in its rapid effects on neuronal function 20 [31]. Non-genomic signaling occurs when estrogen binds to receptors located on the cell membrane, triggering rapid cellular responses such as changes in ion channel activity and modulation of second messenger systems. These rapid effects are particularly important for synaptic plasticity, the strengthening or weakening of synaptic connections that underpins learning and memory [31,32]. Estrogen’s ability to influence these pathways through both genomic and non-genomic mechanisms highlights the complexity of its role in brain function.

The interplay between ERs and other cellular signaling pathways adds another layer of complexity to how estrogen influences brain health. In addition to its effects on neurotransmitter systems and neuroplasticity, estrogen interacts with inflammation and oxidative stress pathways. Both inflammation and oxidative damage are thought to contribute to age-related cognitive decline, and estrogen’s anti-inflammatory and antioxidant properties may help protect the brain from these damaging effects [33]. Studies have shown that estrogen can reduce the levels of pro-inflammatory cytokines in the brain, which may help mitigate the cognitive deficits associated with aging [34]. Furthermore, estrogen has been shown to protect against oxidative stress by increasing the expression of antioxidant enzymes in neurons, offering another neuroprotective mechanism that is diminished during menopause [35].

The decline in estrogen during menopause, therefore, disrupts these intricate mechanisms, leading to a range of cognitive and emotional changes. The reduced activation of ERs in the brain contributes to the onset of symptoms such as memory impairment, difficulty concentrating, and increased susceptibility to mood disorders like anxiety and depression [4]. While the loss of ERs and their functions during menopause is well-established, the complexity of estrogen’s actions in the brain, through genomic and non-genomic signaling, as well as interactions with other molecular pathways, remains an area of active research [36,37].

## 3. Estrogen and Menopausal Mental Health

The menopausal transition, marked by fluctuating estrogen levels prior to the final menstrual period, is often accompanied by psychological and cognitive challenges. In contrast, the postmenopausal stage, defined by the permanent cessation of menstruation, is characterized by a sustained reduction in estrogen. One of the most significant and commonly experienced consequences of estrogen depletion during menopause is its impact on mental health, particularly mood disorders such as depression and anxiety, as well as cognitive decline [38]. Estrogen’s well-documented role in the brain has far-reaching implications for both emotional regulation and cognitive function, making its loss during menopause a critical factor in the emergence of these mental health disturbances [39].

Research has shown that estrogen plays a direct and vital role in regulating mood by modulating neurotransmitter systems that are involved in mood stabilization. The most extensively studied of these neurotransmitters is serotonin, a key regulator of mood, anxiety, and well-being [26]. Estrogen influences serotoninergic pathways by enhancing the expression and sensitivity of serotonin receptors in key regions of the brain, such as the hippocampus and prefrontal cortex. This modulation helps maintain a balanced mood and has an antidepressant-like effect, which is particularly important during menopause, when fluctuations in estrogen levels are at their peak [22]. When estrogen levels drop, as they do in menopause, the resulting decrease in serotonin receptor expression and activity may contribute to the onset of mood disturbances, including depression and anxiety [40].

Menopausal depression is a hormone-sensitive mood disorder that arises specifically during the menopausal transition and early postmenopause. Although it shares features with major depressive disorder (MDD), menopausal depression presents a distinct symptom profile characterized by mood lability, irritability, heightened anxiety, cognitive difficulties such as impaired attention and memory, fatigue, sleep disturbance, and somatic complaints, rather than pervasive sadness alone. To capture these unique features, Kulkarni and colleagues developed and validated the Meno-D rating scale, which includes domains of somatic, cognitive, self, sleep, and sexual function, thereby supporting its validity as a menopause-specific diagnostic tool [41].

**Figure 2:**
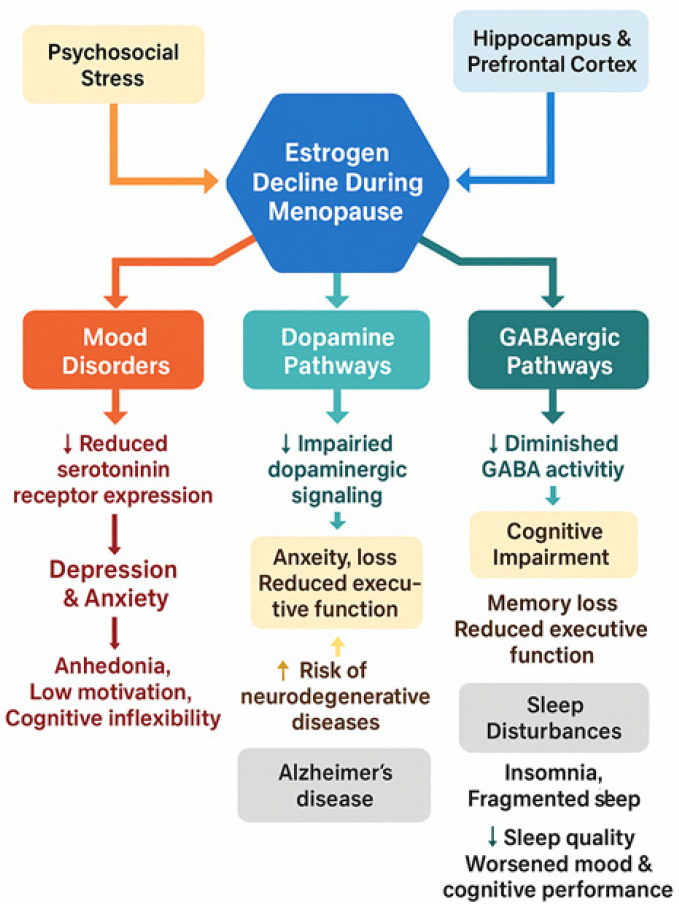
Impact of estrogen decline during menopause on mood, cognition, and mental health. Central role of hormonal fluctuations during the menopausal transition and the sustained estrogen decline after menopause, and their downstream effects on brain function and mental health. Estrogen decline directly impacts neurotransmitter systems, reducing serotonin receptor expression, impairing dopaminergic signaling, and diminishing GABAergic activity, leading to mood disorders such as depression, anxiety, and cognitive dysfunction. The hippocampus and prefrontal cortex, key regions for memory and executive function, are particularly affected, resulting in memory loss and cognitive inflexibility. Additionally, estrogen’s role in sleep regulation is disrupted, contributing to insomnia and poor sleep quality, which further exacerbate emotional and cognitive disturbances. The cumulative effects increase the risk of neurodegenerative diseases, including Alzheimer’s disease. Psychosocial stressors may further intensify these symptoms during the menopausal transition.

The biological underpinnings of menopausal depression involve fluctuating and declining estrogen levels, which modulate serotonergic and dopaminergic neurotransmission. Experimental estradiol-withdrawal studies demonstrate recurrence of depressive symptoms in susceptible women, highlighting the causal role of ovarian hormones in mood regulation [42]. Clinically, hormone-based treatments show promise. Transdermal estradiol combined with intermittent micronized progesterone has been shown to reduce depressive symptoms in early perimenopausal women [43], while a recent pilot trial by Kulkarni et al. reported that bazedoxifene plus conjugated estrogens significantly improved menopausal depression symptoms [44]. These findings underscore the need to differentiate menopausal depression from generic MDD in order to facilitate accurate diagnosis and targeted treatment approaches (Figure 2).

Numerous studies have established a clear link between menopausal estrogen decline and the increased risk of depression in women. This is particularly notable given the fact that women are already more likely to experience depression than men, with menopause marking a period of heightened vulnerability [45,46,47].

In addition to serotonin, estrogen also modulates the activity of other neurotransmitters, such as dopamine and gamma-aminobutyric acid (GABA), which are crucial for emotional regulation and cognitive processes. Dopamine, for example, is involved in motivation, reward processing, and mood regulation [48]. During menopause, the decline in estrogen levels can impair dopaminergic signaling, which may contribute to reduced motivation, feelings of anhedonia, and a general decline in cognitive flexibility [49,50,51]. Dopamine dysregulation has also been implicated in the development of depressive symptoms, and the loss of estrogen’s influence on dopamine receptors can exacerbate this [52]. Similarly, GABA, the primary inhibitory neurotransmitter in the brain, plays a key role in reducing neuronal excitability and anxiety. Estrogen enhances GABAergic activity, which helps counteract the effects of stress and anxiety [53]. As estrogen declines during menopause, GABAergic activity is diminished, which may contribute to the increased anxiety, irritability, and mood swings often reported by women in this phase of life [54].

The link between estrogen deficiency and mood disorders during menopause is further supported by studies showing that women undergoing menopause experience a higher incidence of generalized anxiety disorder (GAD), panic disorder, and other anxiety-related conditions [55]. Anxiety symptoms often emerge or worsen during the perimenopausal period and can be both a result of the hormonal fluctuations as well as a response to the psychosocial stresses associated with aging, such as changes in family dynamics, career, and overall self-identity [41,42,56]. The interplay between fluctuating estrogen levels and the heightened sensitivity of the brain’s anxiety circuits may explain why menopause represents a critical window of vulnerability for anxiety disorders [57,58].

Alongside mood disorders, cognitive decline is another pervasive concern during menopause (Figure 2). While mild cognitive changes such as forgetfulness or difficulty concentrating are common, more significant impairments in memory and executive function are also frequently reported [59]. Estrogen’s neuroprotective effects, particularly in areas of the brain involved in memory, such as the hippocampus, make its loss during menopause a potential trigger for the acceleration of cognitive decline. Research has consistently shown that women undergoing menopause exhibit a decline in verbal memory, attention, and spatial memory, functions that are highly sensitive to estrogen levels [60]. This decline is thought to be mediated by the loss of estrogen’s ability to maintain synaptic plasticity and neurogenesis, both of which are essential for learning and memory. The reduction in estrogen receptor activity in the hippocampus, in particular, is believed to underline this cognitive impairment [18]. As estrogen levels drop, there is a decrease in synaptic connections, a reduction in neurotrophic factor expression, and an overall weakening of the brain’s structural integrity, leading to memory difficulties and reduced cognitive performance [61].

Furthermore, studies have demonstrated a link between low estrogen levels and an increased risk of neurodegenerative diseases, such as Alzheimer’s disease [62]. While the exact mechanisms remain unclear, it is believed that estrogen’s ability to regulate neuro-inflammation, oxidative stress, and neuronal survival may play a critical role in protecting the brain against such conditions. The decline in estrogen during menopause disrupts these protective mechanisms, potentially accelerating the onset of age-related cognitive disorders [63]. Women with a history of early menopause or those who experience a more abrupt decline in estrogen are particularly at risk, with some studies showing that HRT can reduce the risk of developing Alzheimer’s disease when initiated early in the menopausal transition [63].

In addition to depression, anxiety, and cognitive decline, women in menopause may also experience sleep disturbances, which can further exacerbate mental health problems [64]. Estrogen’s effect on the hypothalamus, a region of the brain that regulates circadian rhythms and sleep patterns, is well-documented. A decline in estrogen levels during menopause can lead to disruptions in sleep, particularly in the form of insomnia and fragmented sleep, which are common symptoms of the menopausal transition. These sleep disturbances can lead to increased stress, poor mood regulation, and difficulty concentrating during the day, compounding the negative effects on mental health and cognitive function [65].

## 4. Mechanisms of Estrogen Action on the Brain

**Figure 3:**
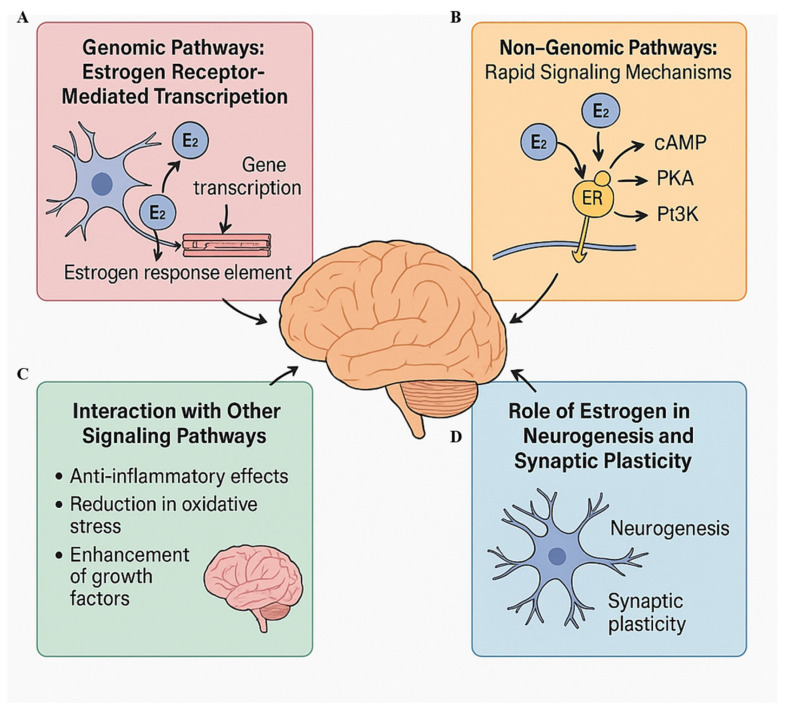
Mechanisms of estrogen action on the brain. multifaceted mechanisms by which estrogen influences brain function. (**A**) Genomic pathways involve estrogen binding to nuclear estrogen receptors (ERα and ERβ), leading to gene transcription via estrogen response elements, promoting synaptic plasticity, neurogenesis, and neuronal survival. (**B**) Non-genomic pathways depict rapid signaling through membrane-bound estrogen receptors activating second messengers such as cyclic adenosine monophosphate (cAMP), protein kinase A (PKA), and phosphatidylinositol-3-kinase (PI3K), enhancing synaptic strength and neuronal excitability. (**C**) Estrogen’s interaction with other signaling pathways includes anti-inflammatory actions, reduction in oxidative stress, and upregulation of growth factors like BDNF. (**D**) Estrogen supports neurogenesis and synaptic plasticity, particularly in the hippocampus, by increasing dendritic spine density and promoting new neuron formation, processes essential for learning, memory, and emotional regulation. ER: estrogen receptor, E2: estradiol.

The actions of estrogen in the brain are mediated through complex mechanisms involving both genomic and non-genomic pathways. These mechanisms highlight the multifaceted roles estrogen plays in maintaining brain function, influencing mood, memory, and cognition, especially in the context of menopause [40]. As estrogen levels decline during menopause, the disruption of these pathways can lead to cognitive impairments and mood disorders. Understanding the molecular and cellular processes by which estrogen affects the brain is crucial for developing effective treatments for menopausal symptoms [59]. The following subsections describe these mechanisms in detail (Figure 3).

### 4.1. Genomic Pathways: Estrogen Receptor-Mediated Transcription

Upon binding to estrogen, these receptors undergo a conformational change that allows them to interact with specific DNA sequences known as estrogen response elements (EREs), located in the promoter regions of target genes. This interaction leads to the activation or repression of gene transcription, which ultimately influences cellular function and survival [66].

In the hippocampus, for example, estrogen binding to ERα and ERβ has been shown to promote the expression of genes involved in synaptic plasticity, neurogenesis, and neuronal survival [67]. Estrogen-induced changes in gene expression result in the production of proteins such as brain-derived neurotrophic factor (BDNF) and other neurotrophins that support neuronal growth and synaptic connectivity. These processes are fundamental for learning, memory, and emotional regulation [68]. Additionally, estrogen has been shown to modulate the expression of ion channels, neurotransmitter receptors, and enzymes involved in neurotransmitter synthesis and metabolism [69,70,71]. Through these genomic actions, estrogen can enhance the brain’s capacity to adapt to new information, maintain cognitive function, and regulate mood [71].

One of the key effects of estrogen on gene transcription is its regulation of neuroplasticity. Estrogen’s influence on genes that govern the formation and strengthening of synapses is crucial for maintaining cognitive health, particularly in regions like the hippocampus that are essential for memory and learning [72]. In animal models, estrogen administration has been shown to increase the number of dendritic spines, structures where synaptic connections are formed [31,73]. These structural changes are associated with improved learning and memory, and their reduction in postmenopausal women may underlie the cognitive decline often observed during this life stage [74]. Beyond these preclinical and mechanistic findings, recent studies have applied non-invasive brain stimulation and psychophysiological methods to investigate estrogen’s role in cognition, including evidence for its influence on multisensory integration [75]. In addition, neuropsychological testing in menopausal women provides converging clinical evidence linking cognitive changes during this transition to hormonal fluctuations.

### 4.2. Non-Genomic Pathways: Rapid Signaling Mechanisms

In addition to its genomic effects, estrogen also exerts rapid, non-genomic actions in the brain. These non-genomic actions occur within minutes to hours after estrogen binding to its receptors, and they do not involve direct changes in gene transcription. Instead, estrogen activates intracellular signaling pathways that lead to immediate alterations in cellular function. ERs, particularly ERα, are localized not only in the nucleus but also on the plasma membrane of neurons, where they can initiate rapid signaling events [76].

One of the key non-genomic actions of estrogen is the activation of second messenger systems, such as cyclic AMP (cAMP), protein kinase A (PKA), and phosphatidylinositol-3-kinase (PI3K) [72,74]. These pathways are involved in various cellular processes, including synaptic transmission, cell survival, and neuronal excitability [73]. For example, estrogen binding to ERα on the cell membrane activates the PKA pathway, which can lead to the phosphorylation of proteins involved in synaptic plasticity. This rapid signaling enhances the strength of synaptic connections and contributes to the dynamic nature of learning and memory [66].

Estrogen also influences intracellular calcium signaling, which is crucial for synaptic function and plasticity. By modulating calcium channels, estrogen can affect the release of neurotransmitters and the activation of signaling cascades that regulate neuronal excitability and synaptic strength. These rapid effects are particularly important in the context of learning, as they enable neurons to respond quickly to new stimuli and experiences [77]. The non-genomic signaling pathways are thought to complement estrogen’s genomic actions, providing a balance between long-term changes in gene expression and immediate adjustments in neuronal activity [73].

### 4.3. Interaction with Other Signaling Pathways

In addition to its direct effects on gene transcription and rapid signaling pathways, estrogen interacts with a variety of other signaling systems that influence brain function. One of the most important of these is the inflammatory response [66]. Estrogen has well-documented anti-inflammatory properties, which are mediated in part by its ability to inhibit the activation of pro-inflammatory cytokines [31,73]. During menopause, the decline in estrogen levels can lead to an increase in neuro-inflammation, which has been associated with cognitive decline, mood disturbances, and the exacerbation of neurodegenerative diseases [78]. Estrogen’s ability to suppress neuro-inflammation helps protect neurons from damage and supports overall brain health [79].

Estrogen also plays a role in oxidative stress, a condition in which there is an excess of reactive oxygen species (ROS) that can damage cells and tissues. Oxidative stress is a major contributor to age-related cognitive decline, and estrogen has been shown to upregulate the expression of antioxidant enzymes such as superoxide dismutase (SOD) and glutathione peroxidase, which help neutralize ROS [80]. By reducing oxidative stress, estrogen protects neurons from damage and supports long-term cognitive function. However, as estrogen levels decline during menopause, this protective effect is diminished, contributing to the increased vulnerability of the brain to oxidative damage [62].

In particular, BDNF, strongly regulated by estrogen, plays a pivotal role in hippocampal synaptic plasticity and memory processes [68]. Estrogen enhances the expression of BDNF and other growth factors, thereby promoting neurogenesis and synaptogenesis. This action is particularly important during menopause, when the decline in estrogen can lead to a reduction in BDNF levels and impair synaptic function. The loss of estrogen’s neurotrophic effects may contribute to the cognitive decline observed in many postmenopausal women [81].

### 4.4. Role of Estrogen in Neurogenesis and Synaptic Plasticity

One of the most significant effects of estrogen in the brain is its role in neurogenesis, the process by which new neurons are generated from neural stem cells. Estrogen has been shown to stimulate neurogenesis in key brain regions involved in learning and memory, particularly the hippocampus [82]. In preclinical models, estrogen treatment has been associated with an increase in the number of new neurons in the hippocampus, a process that is thought to be essential for maintaining cognitive function and emotional regulation [13]. Neurogenesis, particularly in the dentate gyrus of the hippocampus, is crucial for forming new memories and adapting to novel experiences. Estrogen’s ability to promote neurogenesis provides a neuroprotective effect, helping to maintain cognitive abilities and emotional stability during aging [68].

Estrogen also plays a pivotal role in synaptic plasticity, the ability of synapses to strengthen or weaken in response to activity. Estrogen enhances the formation of dendritic spines, which are the sites of synaptic connections between neurons. This increase in dendritic spines improves the capacity for synaptic plasticity, which is essential for learning and memory [13]. The decline in estrogen during menopause, however, leads to a reduction in dendritic spines and synaptic connections, contributing to cognitive decline and memory impairments [13,18,74]. Estrogen’s positive influence on synaptic plasticity and neurogenesis is, therefore, critical for preserving brain function during menopause and aging [63].

## 5. Clinical Implications and Current Therapies

**Figure 4:**
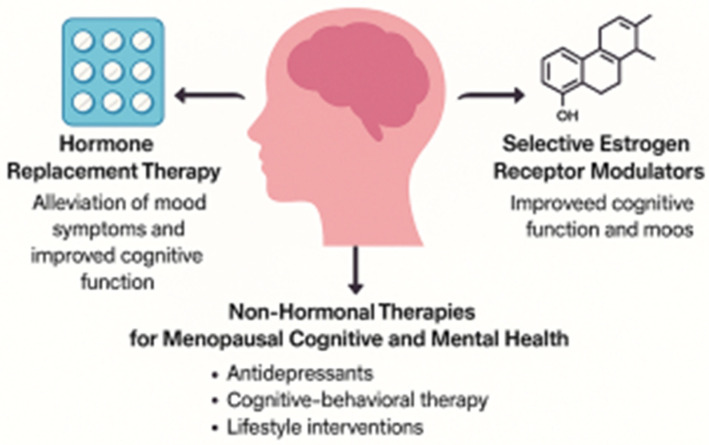
Clinical implications and current therapies for menopausal cognitive and mental health management. Three primary therapeutic approaches for addressing cognitive decline and mood disturbances associated with menopause: Hormone Replacement Therapy (HRT), Selective Estrogen Receptor Modulators (SERMs), and non-hormonal therapies. HRT alleviates mood symptoms and improves cognitive function by restoring estrogen levels, promoting neuroplasticity and neurotransmitter activity. SERMs selectively target estrogen receptors in the brain to provide cognitive and mood benefits while minimizing systemic risks. Non-hormonal approaches, including antidepressants, cognitive-behavioral therapy (CBT), and lifestyle interventions, offer alternative or adjunctive strategies for managing menopausal symptoms without relying on estrogen-based treatments.

Given the significant role estrogen plays in maintaining brain function and mental health, therapeutic strategies aimed at addressing the cognitive and emotional challenges associated with menopause often focus on restoring or mimicking estrogen’s effects in the brain. Estrogen-based therapies, including hormone replacement therapy (HRT) and selective estrogen receptor modulators (SERMs), have been widely studied for their potential to alleviate symptoms such as mood swings, anxiety, depression, and cognitive decline [58]. However, while these treatments have shown promise, their use remains controversial due to concerns about side effects, risks, and long-term safety [59]. This section explores the therapeutic implications of estrogen-based treatments, the challenges they present, and the potential future directions for menopausal cognitive and mental health management (Figure 4).

### 5.1. Hormone Replacement Therapy (HRT)

HRT has been one of the most commonly prescribed treatments for managing the symptoms of menopause, including hot flashes, night sweats, and mood disturbances [63,83]. HRT typically involves the administration of estrogen, either alone or in combination with progestin [84]. Estrogen replacement has been shown to have beneficial effects on mood, reducing the incidence of depressive symptoms and improving overall emotional well-being in many women. In addition to its mood-enhancing effects, estrogen therapy has been linked to improved cognitive function, particularly in the areas of verbal memory, attention, and processing speed [83]. Several clinical studies have suggested that early initiation of HRT, particularly during the perimenopausal period, may provide neuroprotective benefits, reducing the risk of cognitive decline and neurodegenerative diseases such as Alzheimer’s disease [62].

One of the primary mechanisms by which HRT improves cognitive function is by restoring estrogen levels in the brain, thereby promoting neurogenesis and synaptic plasticity. Estrogen replacement enhances the expression of neurotrophic factors such as BDNF, supporting synaptic growth and neuronal survival [13]. In addition, HRT improves neurotransmitter systems including serotonin and dopamine, both of which are central to mood regulation and cognitive performance. By enhancing these systems, estrogen therapy can alleviate mood disorders and improve cognitive flexibility, attention, and memory [63,85].

Despite these positive effects, HRT is not without its risks. Studies have highlighted concerns regarding the potential increased risk of breast cancer, cardiovascular disease, and thromboembolic events associated with long-term HRT use [86]. The Women’s Health Initiative (WHI) study, for instance, raised concerns about the safety of combined estrogen-progestin therapy in postmenopausal women, leading to a decrease in its widespread use [87]. However, it is now recognized that much of the increased risk reported in the WHI was attributable to the use of synthetic progestins rather than estrogen itself. Subsequent re-analyses and recent studies have challenged the initial interpretations of the WHI findings, suggesting that estrogen-alone therapy or regimens using bioidentical progesterone may present a more favorable safety profile [34,87]. Recent research further suggests that when initiated early in the menopausal transition and for limited periods, HRT may offer more favorable risk–benefit profiles, particularly for women who are at a high risk of developing osteoporosis or experiencing severe cognitive decline. As a result, the decision to initiate HRT should be personalized, considering the woman’s age, overall health, and specific menopausal symptoms [88,89].

### 5.2. Selective Estrogen Receptor Modulators (SERMs)

For women who cannot or prefer not to use traditional HRT due to its potential risks, SERMs present an alternative approach. SERMs are compounds that selectively target ERs in specific tissues, such as the brain, bone, and cardiovascular system, providing the benefits of estrogen without some of the associated risks [90]. The most well-known SERM is raloxifene, which has been primarily used for the prevention and treatment of osteoporosis, but research has also suggested its potential to improve cognitive function and mood in menopausal women [91].

Unlike traditional estrogen therapy, which stimulates ERs in multiple tissues, SERMs can act as either agonists or antagonists, depending on the target tissue [92]. In the brain, certain SERMs have been shown to mimic estrogen’s positive effects on synaptic plasticity and neurotransmitter systems without inducing the same level of risk for breast or uterine cancer [93]. For example, compounds like tamoxifen and bazedoxifene have shown promising results in improving cognitive function in postmenopausal women by enhancing neuroplasticity and neurogenesis, particularly in the hippocampus [94,95]. Additionally, SERMs have been associated with improved mood regulation, likely due to their effects on serotonin and dopamine systems [26].

While SERMs offer a more targeted approach to estrogen receptor modulation, they are not without limitations. One of the challenges with SERM therapy is their tissue-specific action, which can lead to varying degrees of effectiveness across different systems [96]. Furthermore, the long-term safety and efficacy of SERMs in managing cognitive decline and mood disorders in postmenopausal women remain an area of active investigation. Although they provide a safer alternative to traditional HRT, more research is needed to establish the optimal use of SERMs for addressing menopausal mental health issues [83,97].

### 5.3. Non-Hormonal Therapies and Lifestyle Interventions

While estrogen-based therapies remain the gold standard for many women, non-hormonal treatments have become increasingly important, particularly for those who cannot or choose not to use HRT or SERMs. Several classes of medications and interventions have shown promise in managing menopausal cognitive and mental health symptoms. These include antidepressants, cognitive-behavioral therapy (CBT), and lifestyle interventions [83].

Antidepressants, particularly selective serotonin reuptake inhibitors (SSRIs) and serotonin-norepinephrine reuptake inhibitors (SNRIs), are often prescribed to manage mood disorders such as depression and anxiety during menopause. SSRIs and SNRIs work by increasing the availability of serotonin and norepinephrine in the brain, both of which play critical roles in mood regulation [98]. These medications can help alleviate depressive symptoms and reduce anxiety, though their effects on cognitive function during menopause remain less clear. They are often used as a first-line treatment for women who cannot take estrogen-based therapies [99].

Cognitive-behavioral therapy (CBT) is another effective non-hormonal intervention. CBT has been shown to improve mood, reduce anxiety, and enhance cognitive functioning in menopausal women. It focuses on changing negative thought patterns and improving coping strategies, which can be particularly helpful for women experiencing the emotional upheaval of menopause. In fact, CBT may provide long-lasting benefits without the side effects associated with pharmacological treatments [100].

Lifestyle interventions, such as exercise, a balanced diet, and adequate sleep, have also demonstrated significant positive effects on both cognitive function and mental health during menopause [101]. Regular physical activity, in particular, has been shown to improve mood, reduce anxiety, and enhance cognitive performance. Exercise can increase serotonin and dopamine levels in the brain, similar to the effects of estrogen, providing a natural way to mitigate some of the cognitive and emotional symptoms of menopause [102]. In addition, maintaining a healthy diet rich in omega-3 fatty acids, antioxidants, and vitamins has been linked to better cognitive health and mood regulation [103].

Finally, combination therapies that incorporate both hormonal and non-hormonal interventions may become more common in the future. For instance, combining HRT with cognitive training, mindfulness practices, or exercise could provide a comprehensive approach to managing menopausal symptoms, addressing both the biological and psychological aspects of the transition.

## 6. Challenges and Gaps in Research

While estrogen-based therapies, such as HRT and SERMs, have demonstrated significant benefits in managing menopausal mental health issues, their use is not without significant challenges and controversies. The decision to initiate estrogen-based treatments must be carefully considered, as these therapies come with a range of potential risks, side effects, and long-term health implications.

### 6.1. Safety Concerns and Risks

One of the primary concerns with estrogen-based therapies, particularly HRT, is the potential risk of breast cancer and cardiovascular events. Large-scale studies, such as the Women’s Health Initiative (WHI), have raised concerns about the increased risk of breast cancer, coronary artery disease, and stroke with the long-term use of combined estrogen-progestin therapies [87,104]. The WHI study, which found a significant association between combined HRT and increased cardiovascular risk, led to a decline in HRT usage in many countries [105]. Although later studies have suggested that the timing of HRT initiation may play a critical role, indicating that starting HRT near the onset of menopause might reduce risks, the overall concerns about its safety in postmenopausal women continue to persist [104].

Furthermore, venous thromboembolism (VTE) is another risk associated with estrogen therapy. Studies have shown that women on estrogen therapy have an increased incidence of blood clots, which can lead to deep vein thrombosis (DVT) or pulmonary embolism. This risk is particularly elevated in women who have other predisposing factors, such as obesity, smoking, or a family history of clotting disorders [106,107].

### 6.2. Personalization of Treatment

Given the diverse risks associated with estrogen therapy, the importance of personalized treatment has gained increasing recognition. Factors such as a woman’s age, the severity of menopausal symptoms, personal and family medical history, and overall health profile must be considered when determining whether to use HRT or other estrogen-based therapies. In particular, younger women or those experiencing early menopause may benefit more from HRT, with fewer associated risks, compared to older women. For women with a history of breast cancer or other estrogen-sensitive cancers, the use of estrogen therapy is often contraindicated, further emphasizing the need for tailored, individualized treatment plans.

The choice between estrogen therapy and alternatives weighing the potential benefits for cognitive and mental health against the associated risks. In some cases, alternative treatments that do not carry the same risks, such as non-hormonal medications or lifestyle modifications, may be more appropriate.

### 6.3. Social and Cultural Factors

Beyond the biological and clinical concerns, there are also significant social and cultural factors that influence the decision to use estrogen therapies. For many women, the use of HRT can feel stigmatized, with societal pressures to either “embrace” menopause or seek medical interventions. Cultural perceptions around aging, women’s health, and the stigmatization of menopause symptoms may discourage women from seeking treatment or discussing their symptoms openly with healthcare providers. In some cultures, menopause is seen as a natural and unavoidable part of aging, which leads to reluctance to pursue pharmacological treatments. Education, awareness, and open communication about the benefits and risks of estrogen therapies are crucial in empowering women to make informed decisions regarding their treatment options.

### 6.4. Unanswered Questions and Research Gaps

Despite the promising effects of estrogen-based therapies, there remain several unanswered questions and critical research gaps that must be addressed. For instance, the long-term effects of estrogen therapy on brain health, especially with regard to Alzheimer’s disease, remain unclear. While some studies suggest that HRT initiated early in menopause may provide neuroprotective benefits, others show inconsistent results [108]. Furthermore, the specific mechanisms by which estrogen influences brain function in the context of menopause, especially in terms of how different ERs mediate these effects, are still not fully understood. Future research is essential to address these gaps, optimizing estrogen therapies and their impact on mental and cognitive health in menopausal women.

## 7. Future Directions

As we continue to explore the relationship between ERs and menopausal cognitive and mental health, several exciting avenues for future research emerge. One of the most promising areas involves the development of more targeted estrogen-based therapies. While SERMs have shown some benefits, their ability to effectively cross the blood–brain barrier and target estrogen receptor subtypes like ERβ in the brain is still limited. Future research could focus on designing new compounds that better mimic estrogen’s neuroprotective effects without the risks associated with traditional HRT, especially regarding reproductive tissues like the breast and uterus. Additionally, the role of estrogen receptor subtypes, particularly ERα and ERβ, in various brain regions remains an area of active investigation. Understanding how these receptors function in specific areas such as the hippocampus, amygdala, and prefrontal cortex could provide valuable insights into their distinct roles in regulating cognition, mood, and neuroprotection.

Another critical area for future exploration is the timing and duration of HRT. Evidence suggests that initiating HRT early in menopause may offer neuroprotective benefits, but the risks associated with long-term use remain a concern. Longitudinal studies that examine the optimal window for HRT initiation, its impact on cognitive function, and the most effective duration of therapy will be crucial in refining clinical guidelines. Moreover, as research into non-hormonal therapies grows, there is increasing interest in understanding how antidepressants, CBT, and lifestyle interventions can complement estrogen-based treatments. The synergy between hormonal and non-hormonal therapies could provide a more holistic approach to managing menopausal mental health and cognitive decline.

Genetic and epigenetic factors also hold promise for personalizing treatment. Understanding how genetic variations influence a woman’s response to estrogen therapies or her susceptibility to cognitive decline during menopause will pave the way for individualized approaches to treatment. Epigenetic changes may also play a role in how environmental factors and lifestyle choices influence menopausal outcomes. Furthermore, the connection between estrogen loss and neurodegenerative diseases such as Alzheimer’s disease remains an important area for research. Estrogen’s neuroprotective properties could help delay or prevent neurodegeneration, particularly in women at higher genetic risk. Finally, future studies should explore estrogen’s broader impact on other brain functions, including pain perception, sleep regulation, and appetite control, which are often affected during menopause. With these advancements, it is likely that more effective, targeted, and safer treatments will emerge, offering menopausal women better options for managing the mental and cognitive challenges associated with this life stage.

## 8. Conclusions

Menopause marks a pivotal transition in a woman’s life, with a significant impact on mental and cognitive health due to the decline in estrogen levels. While estrogen-based therapies like HRT and SERMs show promise in alleviating mood disorders and cognitive decline, their use must be carefully tailored to each woman’s individual health profile. The risks associated with long-term use of these therapies, particularly regarding cancer and cardiovascular health, require a personalized approach to treatment.

The present review highlights the strengths of providing a comprehensive synthesis of evidence on the role of estrogens and estrogen receptors in cognitive and mental health outcomes during menopause, integrating both preclinical and clinical findings. However, some limitations should be acknowledged. As a narrative review, quantitative synthesis was not performed, and the potential for publication bias and study heterogeneity may limit the generalizability of the conclusions. Future systematic reviews and longitudinal studies are warranted to address these gaps.

Looking ahead, future research is crucial to further understand the precise mechanisms by which estrogen impacts brain function and to develop safer, more effective therapies. The integration of targeted estrogen-based treatments, non-hormonal therapies, and lifestyle modifications could provide a comprehensive solution to managing the cognitive and emotional challenges of menopause. Continued investigation into genetic and epigenetic factors, as well as the development of early intervention strategies, holds the potential to significantly improve outcomes for women navigating this life stage.

## Data Availability

No new data were created or analyzed in this study. Data sharing is not applicable to this article.

## Supplementary Materials

The following supporting information can be downloaded at https://www.mdpi.com/article/10.3390/brainsci15091003/s1, Figure S1: PRISMA flow diagram illustrating the study selection process.

## Abbreviations

The following abbreviations are used in this manuscript:

ERs	Estrogen receptors
ERα	Estrogen receptor alpha
ERβ	Estrogen receptor beta
HRT	Hormone replacement therapy
GABA	Gamma-aminobutyric acid
GDA	Generalized anxiety disorder
EREs	Estrogen response elements
BDNF	Brain-derived neurotrophic factor
cAMP	Cyclic adenosine monophosphate
PKA	Protein kinase A
PI3K	Phosphatidylinositol-3-kinase
ROS	Reactive oxygen species
SOD	Superoxide dismutase
WHI	Women’s health initiative
SERMs	Selective estrogen receptor modulators
CBT	Cognitive-behavioral therapy
SSRIs	Selective serotonin reuptake inhibitors
SNRIs	Serotonin-norepinephrine reuptake inhibitors
VTE	Venous thromboembolism
DVT	Deep vein thrombosis

## References

[1] Gold E.B. (2011). The timing of the age at which natural menopause occurs. Obstet. Gynecol. Clin. N. Am..

[2] Caldwell J.Z.K., Isenberg N. (2023). The aging brain: Risk factors and interventions for long term brain health in women. Curr. Opin. Obstet. Gynecol..

[3] Iqbal J., Huang G.D., Xue Y.X., Yang M., Jia X.J. (2024). Role of estrogen in sex differences in memory, emotion and neuropsychiatric disorders. Mol. Biol. Rep..

[4] Osterlund M.K., Hurd Y.L. (2001). Estrogen receptors in the human forebrain and the relation to neuropsychiatric disorders. Prog. Neurobiol..

[5] Almey A., Milner T.A., Brake W.G. (2015). Estrogen receptors in the central nervous system and their implication for dopamine-dependent cognition in females. Horm. Behav..

[6] Shughrue P.J., Lane M.V., Merchenthaler I. (1997). Comparative distribution of estrogen receptor-α and -β mRNA in the rat central nervous system. J. Comp. Neurol..

[7] Baek D.C., Kang J.Y., Lee J.S., Lee E.J., Son C.G. (2024). Linking alterations in estrogen receptor expression to memory deficits and depressive behavior in an ovariectomy mouse model. Sci. Rep..

[8] Meng Q., Chao Y., Zhang S., Ding X., Feng H., Zhang C., Liu B., Zhu W., Li Y., Zhang Q. (2023). Attenuation of estrogen and its receptors in the post-menopausal stage exacerbates dyslipidemia and leads to cognitive impairment. Mol. Brain.

[9] Hwang W.J., Lee T.Y., Kim N.S., Kwon J.S. (2020). The Role of Estrogen Receptors and Their Signaling across Psychiatric Disorders. Int. J. Mol. Sci..

[10] Sato K., Takayama K.I., Inoue S. (2023). Expression and function of estrogen receptors and estrogen-related receptors in the brain and their association with Alzheimer’s disease. Front. Endocrinol..

[11] González M., Cabrera-Socorro A., Pérez-García C.G., Fraser J.D., López F.J., Alonso R., Meyer G. (2007). Distribution patterns of estrogen receptor α and β in the human cortex and hippocampus during development and adulthood. Cereb. Cortex.

[12] Tanapat P., Hastings N.B., Reeves A.J., Gould E. (1999). Estrogen stimulates a transient increase in the number of new neurons in the dentate gyrus of the adult female rat. J. Neurosci..

[13] Hara Y., Waters E.M., McEwen B.S., Morrison J.H. (2015). Estrogen Effects on Cognitive and Synaptic Health Over the Lifecourse. Physiol. Rev..

[14] Numpang B., Ke X., Yu X., Callaway C., McKnight R., Joss-Moore L., Lane R. (2013). Fetal growth restriction alters hippocampal 17-beta estradiol and estrogen receptor alpha levels in the newborn male rat. Syst. Biol. Reprod. Med..

[15] Galea L.A.M. (2008). Gonadal hormone modulation of neurogenesis in the dentate gyrus of the adult hippocampus. Front. Neuroendocrinol..

[16] Mazzucco C.A., Lieblich S.E., Bingham B.I., Williamson M.A., Viau V., Galea L.A.M. (2006). Both estrogen receptor α and estrogen receptor β agonists enhance cell proliferation in the dentate gyrus of adult female rats. Neuroscience.

[17] Woolley C.S. (2007). Estrogen-mediated structural and functional synaptic plasticity in the female brain. Horm. Behav..

[18] Flannery J.C., Tirrell P.S., Baumgartner N.E., Daniel J.M. (2025). Neuroestrogens, the hippocampus, and female cognitive aging. Horm. Behav..

[19] Nerattini M., Jett S., Andy C., Carlton C., Zarate C., Boneu C., Battista M., Pahlajani S., Loeb-Zeitlin S., Havryulik Y. (2023). Systematic review and meta-analysis of the effects of menopause hormone therapy on risk of Alzheimer’s disease and dementia. Front. Aging Neurosci..

[20] Shansky R.M. (2009). Estrogen, stress, and the prefrontal cortex: Towards a balanced view. Horm. Behav..

[21] Bethea C.L., Lu N.Z., Gundlah C., Streicher J.M. (2002). Diverse actions of ovarian steroids in the serotonin neural system. Front. Neuroendocrinol..

[22] Mosconi L., Nerattini M., Matthews D.C., Jett S., Andy C., Williams S., Yepez C.B., Zarate C., Carlton C., Fauci F. (2024). In vivo brain estrogen receptor density by neuroendocrine aging and relationships with cognition and symptomatology. Sci. Rep..

[23] Shanmugan S., Epperson C.N. (2014). Estrogen and the prefrontal cortex: Towards a new understanding of estrogen’s effects on executive functions in the menopause transition. Hum. Brain Mapp..

[24] Rubinow D.R., Schmidt P.J. (2019). Sex differences and the neurobiology of affective disorders. Neuropsychopharmacology.

[25] Österlund M.K., Overstreet D.H., Hurd Y.L. (1999). The estrogen receptor β agonist diarylpropionitrile increases serotonin transporter binding in the rat brain. Brain Res..

[26] Bendis P.C., Zimmerman S., Onisiforou A., Zanos P., Georgiou P. (2024). The impact of estradiol on serotonin, glutamate, and dopamine systems. Front. Neurosci..

[27] Zhang Y., Tan X., Tang C. (2024). Estrogen-immuno-neuromodulation disorders in menopausal depression. J. Neuroinflamm..

[28] Yoest K.E., Cummings J.A., Becker J.B. (2014). Estradiol, Dopamine and Motivation. Cent. Nerv. Syst. Agents Med. Chem..

[29] Eck S.R., Bangasser D.A. (2020). The effects of early life stress on motivated behaviors: A role for gonadal hormones. Neurosci. Biobehav. Rev..

[30] Kim D.I., Park Y.M. (2022). Effects of Menopause on Physical Activity and Dopamine Signaling in Women. Iran. J. Public Health.

[31] Raz L., Khan M.M., Mahesh V.B., Vadlamudi R.K., Brann D.W. (2008). Rapid estrogen signaling in the brain. Neurosignals.

[32] Ogiue-Ikeda M., Tanabe N., Mukai H., Hojo Y., Murakami G., Tsurugizawa T., Takata N., Kimoto T., Kawato S. (2008). Rapid modulation of synaptic plasticity by estrogens as well as endocrine disrupters in hippocampal neurons. Brain Res. Rev..

[33] Cheng Y.J., Lin C.H., Lane H.Y. (2021). From Menopause to Neurodegeneration—Molecular Basis and Potential Therapy. Int. J. Mol. Sci..

[34] Sárvári M., Hrabovszky E., Kalló I., Solymosi N., Tóth K., Likó I., Széles J., Mahó S., Molnár B., Liposits Z. (2011). Estrogens regulate neuroinflammatory genes via estrogen receptors α and β in the frontal cortex of middle-aged female rats. J. Neuroinflamm..

[35] Razmara A., Duckles S.P., Krause D.N., Procaccio V. (2007). Estrogen suppresses brain mitochondrial oxidative stress in female and male rats. Brain Res..

[36] Tecalco-Cruz A.C., López-Canovas L., Azuara-Liceaga E. (2023). Estrogen signaling via estrogen receptor alpha and its implications for neurodegeneration associated with Alzheimer’s disease in aging women. Metab. Brain Dis..

[37] Maioli S., Leander K., Nilsson P., Nalvarte I. (2021). Estrogen receptors and the aging brain. Essays Biochem..

[38] Soares C.N. (2023). Menopause and Mood: The Role of Estrogen in Midlife Depression and Beyond. Psychiatr. Clin. North Am..

[39] Hogervorst E., Craig J., O’Donnell E. (2022). Cognition and mental health in menopause: A review. Best. Pract. Res. Clin. Obstet. Gynaecol..

[40] Leng G., Leng R.I. (2021). Oxytocin: A citation network analysis of 10 000 papers. J. Neuroendocrinol..

[41] Kulkarni J., Gavrilidis E., Hudaib A.R., Bleeker C., Worsley R., Gurvich C. (2018). Development and Validation of a New Rating Scale for Perimenopausal Depression—The Meno-D. Transl. Psychiatry.

[42] Schmidt P.J., Ben Dor R., Martinez P.E., Guerrieri G.M., Harsh V.L., Thompson K., Koziol D.E., Nieman L.K., Rubinow D.R. (2015). Effects of Estradiol Withdrawal on Mood in Women with Past Perimenopausal Depression: A Randomized Clinical Trial. JAMA Psychiatry.

[43] Cohen L.S., Soares C.N., Vitonis A.F., Otto M.W., Harlow B.L. (2006). Risk for new onset of depression during the menopausal transition: The Harvard study of moods and cycles. Arch. Gen. Psychiatry.

[44] Bromberger J.T., Kravitz H.M., Matthews K., Youk A., Brown C., Feng W. (2009). Major depression during and after the menopausal transition: Study of Women’s Health Across the Nation (SWAN). Psychol. Med..

[45] Freeman E.W., Sammel M.D., Lin H., Nelson D.B. (2006). Associations of hormones and menopausal status with depressed mood in women with no history of depression. Arch. Gen. Psychiatry.

[46] Becker J.B. (1999). Gender differences in dopaminergic function in striatum and nucleus accumbens. Pharmacol. Biochem. Behav..

[47] Yoest K.E., Quigley J.A., Becker J.B. (2018). Rapid effects of ovarian hormones in dorsal striatum and nucleus accumbens. Horm. Behav..

[48] Arevalo M.-A., Azcoitia I., Garcia-Segura L.M. (2015). The neuroprotective actions of oestradiol and oestrogen receptors. Nat. Rev. Neurosci..

[49] Schultz W. (2007). Multiple dopamine functions at different time courses. Annu. Rev. Neurosci..

[50] Bromberger J.T., Kravitz H.M., Chang Y., Randolph J.F., Avis N.E., Gold E.B., Matthews K.A. (2013). Does Risk for Anxiety Increase During the Menopausal Transition? Study of Women’s Health Across the Nation (SWAN). Menopause.

[51] Hantsoo L., Epperson C.N. (2017). Anxiety Disorders Among Women: A Female Lifespan Approach. Focus.

[52] Maran M., Friederici A.D., Zaccarella E. (2022). Syntax through the looking glass: A review on two-word linguistic processing across behavioral, neuroimaging and neurostimulation studies. Neurosci. Biobehav. Rev..

[53] Deng W., Li R. (2015). Juxtamembrane contribution to transmembrane signaling. Biopolymers..

[54] Alblooshi S., Taylor M., Gill N. (2023). Does menopause elevate the risk for developing depression and anxiety? Results from a systematic review. Australas. Psychiatry.

[55] Huang S., Wang Z., Zheng D., Liu L. (2023). Anxiety disorder in menopausal women and the intervention efficacy of mindfulness-based stress reduction. Am. J. Transl. Res..

[56] Soares C.N., Frey B.N. (2010). Challenges and Opportunities to Manage Depression and Anxiety in Midlife Women. Psychiatr. Clin. North Am..

[57] Deshpande N., Rao T.S.S. (2025). Psychological Changes at Menopause: Anxiety, Mood Swings, and Sexual Health in the Biopsychosocial Context. J. Psychosex. Health.

[58] Uçan Yamaç S., Bakir N. (2025). Aging anxiety levels of women in menopause and associated factors. Rev. Assoc. Med. Bras..

[59] Conde D.M., Verdade R.C., Valadares A.L.R., Mella L.F.B., Pedro A.O., Costa-Paiva L. (2021). Menopause and cognitive impairment: A narrative review of current knowledge. World J. Psychiatry.

[60] Metcalf C.A., Duffy K.A., Page C.E., Novick A.M. (2023). Cognitive Problems in Perimenopause: A Review of Recent Evidence. Curr. Psychiatry Rep..

[61] Zárate S., Stevnsner T., Gredilla R. (2017). Role of Estrogen and Other Sex Hormones in Brain Aging. Neuroprotection and DNA Repair. Front. Aging Neurosci..

[62] Ali N., Sohail R., Jaffer S.R., Siddique S., Kaya B., Atowoju I., Imran A., Wright W., Pamulapati S., Choudhry F. (2023). The Role of Estrogen Therapy as a Protective Factor for Alzheimer’s Disease and Dementia in Postmenopausal Women: A Comprehensive Review of the Literature. Cureus.

[63] Mervosh N., Devi G. (2025). Estrogen, menopause, and Alzheimer’s disease: Understanding the link to cognitive decline in women. Front. Mol. Biosci..

[64] Zhang F., Cheng L. (2024). Association between sleep duration and depression in menopausal women: A population-based study. Front. Endocrinol..

[65] Brown A.M.C., Gervais N.J. (2020). Role of Ovarian Hormones in the Modulation of Sleep in Females Across the Adult Lifespan. Endocrinology.

[66] Fuentes N., Silveyra P. (2019). Estrogen receptor signaling mechanisms. Adv. Protein Chem. Struct. Biol..

[67] Sahab-Negah S., Hajali V., Moradi H.R., Gorji A. (2020). The Impact of Estradiol on Neurogenesis and Cognitive Functions in Alzheimer’s Disease. Cell Mol. Neurobiol..

[68] Deb P., Chini A., Guha P., Rishi A., Bhan A., Brady B., Perrotti L.I., Mandal S.S. (2024). Dynamic regulation of BDNF gene expression by estradiol and lncRNA HOTAIR. Gene.

[69] Gordon J.L., Rubinow D.R., Eisenlohr-Moul T.A., Xia K., Schmidt P.J., Girdler S.S. (2018). Efficacy of Transdermal Estradiol and Micronized Progesterone in the Prevention of Depressive Symptoms in the Menopause Transition: A Randomized Clinical Trial. JAMA Psychiatry.

[70] Kulkarni J., Mu E., Li Q., Malicka M., Gavrilidis E., de Castella A., Gurvich C. (2025). Bazedoxifene plus Conjugated Estrogen to Treat Menopausal Depression—A Pilot Study. J. Pharmacol. Exp. Ther..

[71] Krolick K.N., Zhu Q., Shi H. (2018). Effects of Estrogens on Central Nervous System Neurotransmission: Implications for Sex Differences in Mental Disorders. Prog. Mol. Biol. Transl. Sci..

[72] Frick K.M., Tuscher J.J., Koss W.A., Kim J., Taxier L.R. (2018). Estrogenic regulation of memory consolidation: A look beyond the hippocampus, ovaries, and females. Physiol. Behav..

[73] Lai Y.J., Yu D., Zhang J.H., Chen G.J. (2017). Cooperation of Genomic and Rapid Nongenomic Actions of Estrogens in Synaptic Plasticity. Mol. Neurobiol..

[74] Wolcott N.S., Redman W.T., Karpinska M., Jacobs E.G., Goard M.J. (2025). The estrous cycle modulates hippocampal spine dynamics, dendritic processing, and spatial coding. Neuron.

[75] Maccora S., Bolognini N., Mannina C., Torrente A., Agnello L., Lo Sasso B., Ciaccio M., Sireci G., Brighina F. (2023). The Effects of Estradiol Levels on Crossmodal Perception: A Study on the Sound-Induced Flash Illusion in Healthy and Menstrually Related Migraine Individuals. Neurol. Sci..

[76] Lymer J.M., Sheppard P.A.S., Kuun T., Blackman A., Jani N., Mahbub S., Choleris E. (2018). Estrogens and their receptors in the medial amygdala rapidly facilitate social recognition in female mice. Psychoneuroendocrinology.

[77] Brann D.W., Lu Y., Wang J., Zhang Q., Thakkar R., Sareddy G.R., Pratap U.P., Tekmal R.R., Vadlamudi R.K. (2022). Brain-derived estrogen and neural function. Neurosci. Biobehav. Rev..

[78] Harding A.T., Heaton N.S. (2022). The Impact of Estrogens and Their Receptors on Immunity and Inflammation during Infection. Cancers.

[79] Villa A., Vegeto E., Poletti A., Maggi A. (2016). Estrogens, Neuroinflammation, and Neurodegeneration. Endocr. Rev..

[80] Kumar S., Lata K., Mukhopadhyay S., Mukherjee T.K. (2010). Role of estrogen receptors in pro-oxidative and anti-oxidative actions of estrogens: A perspective. Biochim. Biophys. Acta..

[81] Zhang Z., He Z., Pan J., Yuan M., Lang Y., Wei X., Zhang C. (2024). The interaction of BDNF with estrogen in the development of hypertension and obesity, particularly during menopause. Front. Endocrinol..

[82] Huang Y., Sun W., Gao F., Ma H., Yuan T., Liu Z., Liu H., Hu J., Bai J., Zhang X. (2023). Brain-Derived Estrogen Regulates Neurogenesis, Learning and Memory with Aging in Female Rats. Biology.

[83] Sun Q., Li G., Zhao F., Dong M., Xie W., Liu Q., Yang W., Cui R. (2024). Role of estrogen in treatment of female depression. Aging.

[84] Davis S.R., Pinkerton J., Santoro N., Simoncini T. (2023). Menopause—Biology, consequences, supportive care, and therapeutic options. Cell.

[85] Mu E., Chiu L., Kulkarni J. (2025). Using estrogen and progesterone to treat premenstrual dysphoric disorder, postnatal depression and menopausal depression. Front. Pharmacol..

[86] Hodis H.N., Mack W.J. (2022). Menopausal Hormone Replacement Therapy and Reduction of All-Cause Mortality and Cardiovascular Disease: It Is About Time and Timing. Cancer J..

[87] Manson J.E., Chlebowski R.T., Stefanick M.L., Aragaki A.K., Rossouw J.E., Prentice R.L., Anderson G., Howard B.V., Thomson C.A., LaCroix A.Z. (2013). Menopausal hormone therapy and health outcomes during the intervention and extended poststopping phases of the Women’s Health Initiative randomized trials. JAMA.

[88] Na Z., Wei W., Xu Y., Li D., Yin B., Gu W. (2023). Role of menopausal hormone therapy in the prevention of postmenopausal osteoporosis. Open Life Sci..

[89] Wu M., Li M., Yuan J., Liang S., Chen Z., Ye M., Ryan P.M., Clark C., Tan S.C., Rahmani J. (2020). Postmenopausal Hormone Therapy and Alzheimer’s Disease, Dementia, and Parkinson’s Disease: A Systematic Review and Time-Response Meta-Analysis. Pharmacol. Res..

[90] Farkas S., Szabó A., Hegyi A.E., Török B., Fazekas C.L., Ernszt D., Kovács T., Zelena D. (2022). Estradiol and Estrogen-like Alternative Therapies in Use: The Importance of the Selective and Non-Classical Actions. Biomedicines.

[91] Veenman L. (2020). Raloxifene as Treatment for Various Types of Brain Injuries and Neurodegenerative Diseases: A Good Start. Int. J. Mol. Sci..

[92] Pinkerton J.V., Thomas S. (2014). Use of SERMs for treatment in postmenopausal women. J. Steroid Biochem. Mol. Biol..

[93] Russell J.K., Jones C.K., Newhouse P.A. (2019). The Role of Estrogen in Brain and Cognitive Aging. Neurotherapeutics.

[94] Newhouse P., Albert K., Astur R., Johnson J., Naylor M., Dumas J. (2013). Tamoxifen improves cholinergically modulated cognitive performance in postmenopausal women. Neuropsycho-pharmacology.

[95] Hill R.A., Kouremenos K., Tull D., Maggi A., Schroeder A., Gibbons A., Kulkarni J., Sun-dram S., Du X. (2020). Bazedoxifene—A promising brain active SERM that crosses the blood brain barrier and enhances spatial memory. Psychoneuroendocrinology.

[96] Martinkovich S., Shah D., Planey S.L., Arnott J.A. (2014). Selective estrogen receptor modulators: Tissue specificity and clinical utility. Clin. Interv. Aging.

[97] Mills Z.B., Faull R.L.M., Kwakowsky A. (2023). Is Hormone Replacement Therapy a Risk Factor or a Therapeutic Option for Alzheimer’s Disease?. Int. J. Mol. Sci..

[98] Stubbs C., Mattingly L., Crawford S.A., Wickersham E.A., Brockhaus J.L., McCarthy L.H. (2017). Do SSRIs and SNRIs reduce the frequency and/or severity of hot flashes in menopausal women. J. Okla. State Med. Assoc..

[99] Garg R., Munshi A. (2025). Menopause and Mental Health. J. Midlife Health.

[100] Hunter M.S., Chilcot J. (2021). Is cognitive behaviour therapy an effective option for women who have troublesome menopausal symptoms?. Br. J. Health Psychol..

[101] Garg R., Munshi A. (2024). Sleep and Brain Function at Menopause. J. Midlife Health.

[102] Hossain M.N., Lee J., Choi H., Kwak Y.S., Kim J. (2024). The impact of exercise on depression: How moving makes your brain and body feel better. Phys. Act. Nutr..

[103] Dighriri I.M., Alsubaie A.M., Hakami F.M., Hamithi D.M., Alshekh M.M., Khobrani F.A., Dalak F.E., Hakami A.A., Alsueaadi E.H., Alsaawi L.S. (2022). Effects of Omega-3 Polyunsaturated Fatty Acids on Brain Functions: A Systematic Review. Cureus.

[104] Mehta J., Kling J.M., Manson J.E. (2021). Risks, Benefits, and Treatment Modalities of Menopausal Hormone Therapy: Current Concepts. Front. Endocrinol..

[105] Cagnacci A., Venier M. (2019). The Controversial History of Hormone Replacement Therapy. Medicina.

[106] Abou-Ismail M.Y., Citla Sridhar D., Nayak L. (2020). Estrogen and thrombosis: A bench to bedside review. Thromb. Res..

[107] Canonico M., Oger E., Conard J., Meyer G., Lévesque H., Trillot N., Barrellier M.T., Wahl D., Emmerich J., Scarabin P.Y. (2006). Obesity and risk of venous thromboembolism among postmenopausal women: Differential impact of hormone therapy by route of estrogen administration. The ESTHER Study. J. Thromb. Haemost..

[108] Saleh R.N.M., Hornberger M., Ritchie C.W., Minihane A.M. (2023). Hormone replacement therapy is associated with improved cognition and larger brain volumes in at-risk APOE4 women: Results from the European Prevention of Alzheimer’s Disease (EPAD) cohort. Alzheimers Res. Ther..

