# Supplementary material for: Beyond Hot Flashes: The Role of Estrogen Receptors in Menopausal Mental Health and Cognitive Decline
Source: Brain Sci. 2025 Sep 16;15(9):1003. doi: 10.3390/brainsci15091003 (PMC12469143; doi:10.3390/brainsci15091003)
Supplement: Supplementary file 1 [file brainsci-15-01003-s001.zip › brainsci-3855415-supplementary.pptx]

## Slide 1
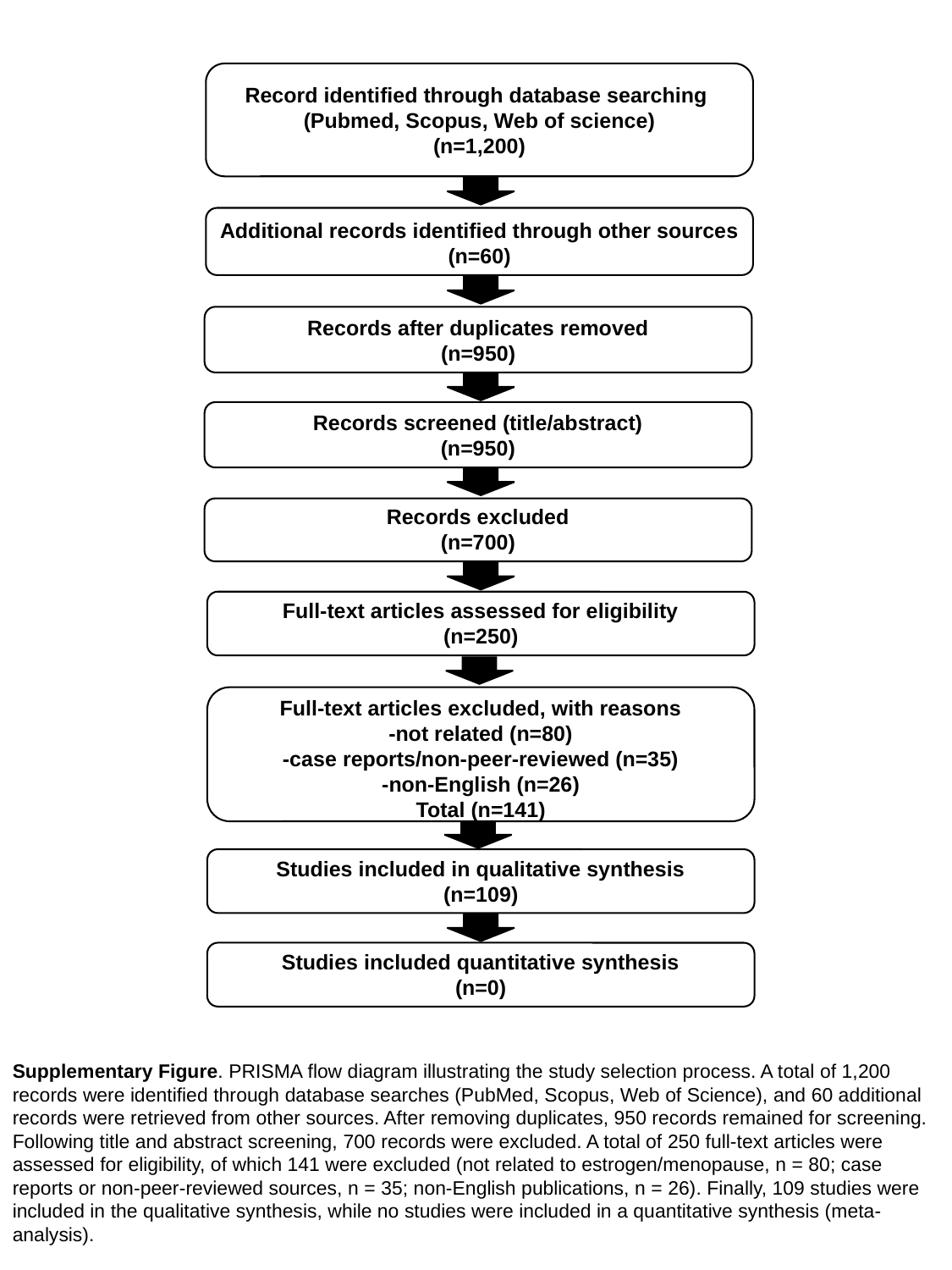

Record identified through database searching
(Pubmed, Scopus, Web of science)
(n=1,200)
Additional records identified through other sources
(n=60)
Records after duplicates removed
(n=950)
Records screened (title/abstract)
(n=950)
Records excluded
(n=700)
Full-text articles assessed for eligibility
(n=250)
Full-text articles excluded, with reasons
-not related (n=80)
-case reports/non-peer-reviewed (n=35)
-non-English (n=26)
Total (n=141)
Studies included in qualitative synthesis
(n=109)
Studies included quantitative synthesis
(n=0)
Supplementary Figure. PRISMA flow diagram illustrating the study selection process. A total of 1,200 records were identified through database searches (PubMed, Scopus, Web of Science), and 60 additional records were retrieved from other sources. After removing duplicates, 950 records remained for screening. Following title and abstract screening, 700 records were excluded. A total of 250 full-text articles were assessed for eligibility, of which 141 were excluded (not related to estrogen/menopause, n = 80; case reports or non-peer-reviewed sources, n = 35; non-English publications, n = 26). Finally, 109 studies were included in the qualitative synthesis, while no studies were included in a quantitative synthesis (meta-analysis).
